# Phenotypic Plasticity: What Has DNA Methylation Got to Do with It?

**DOI:** 10.3390/insects13020110

**Published:** 2022-01-19

**Authors:** Elizabeth J. Duncan, Christopher B. Cunningham, Peter K. Dearden

**Affiliations:** 1 School of Biology, Faculty of Biological Sciences, University of Leeds, Leeds LS2 9JT, UK; 2 Department of Entomology, University of Georgia, Athens, GA 30602, USA; cbc83@uga.edu; 3 Genomics Aotearoa and Department of Biochemistry, University of Otago, Dunedin 9054, New Zealand

**Keywords:** behaviour, development, DNA methylation, epigenetics, phenotypic plasticity

## Abstract

**Simple Summary:**

Phenotypic plasticity, the genome producing multiple phenotypes, is central to an animal’s ability to respond to environmental change, expected or otherwise. A prominent example of this is the behavioural maturation of a honey bee worker over its lifetime. These multiple phenotype outcomes are based on changes in gene expression precipitated by internal and external signals. How these signals are translated from the environment into changes in gene expression is an active area of research. One avenue of investigation has been the responsiveness of DNA methylation, chemical modifications of cytosines, to environmental changes and its influence on gene expression. We review this active field of research and find that cytosine methylation is often altered by environmental changes. However, we find no strong, broad links between changes in cytosine methylation and changes in gene expression. Although there is some evidence that species-specific links between the two occurs. While this is currently the case, we also do not believe the field has arrived at a conclusive answer. Better experimental designs, appropriate biological replication, newer computational tools, and, most importantly, the use of genetic manipulations will provide definitive answers as to the link between phenotypic plasticity and DNA.

**Abstract:**

How does one genome give rise to multiple, often markedly different, phenotypes in response to an environmental cue? This phenomenon, known as phenotypic plasticity, is common amongst plants and animals, but arguably the most striking examples are seen in insects. Well-known insect examples include seasonal morphs of butterfly wing patterns, sexual and asexual reproduction in aphids, and queen and worker castes of eusocial insects. Ultimately, we need to understand how phenotypic plasticity works at a mechanistic level; how do environmental signals alter gene expression, and how are changes in gene expression translated into novel morphology, physiology and behaviour? Understanding how plasticity works is of major interest in evolutionary-developmental biology and may have implications for understanding how insects respond to global change. It has been proposed that epigenetic mechanisms, specifically DNA methylation, are the key link between environmental cues and changes in gene expression. Here, we review the available evidence on the function of DNA methylation of insects, the possible role(s) for DNA methylation in phenotypic plasticity and also highlight key outstanding questions in this field as well as new experimental approaches to address these questions.

Insects are critical to all terrestrial ecosystems: cycling nutrients, dispersing seeds, maintaining soil structure and, importantly, pollinating plants including food crops. Insect biodiversity and abundance are declining across the globe due to the unprecedented challenges of global climate change, habitat loss, disease and pesticide usage (reviewed in [1]). Insects, like most animals, adapt to expected and structured modifications in their environment, such as seasonal weather changes. However, unpredictable or abrupt changes in the environment, such as the extreme temperature fluctuations associated with the current rapid changes in our climate [2,3], can cause significant challenges for these species. Insects may be exquisitely sensitive to environmental cues, such as temperature (they are ectothermic) and nutrition. Such rapid changes may be too fast for classic selection responses and thus rely on phenotypic plasticity. Understanding plastic mechanisms, ecology and evolution of environmental responsiveness and adaptation to new environments in this clade is crucially important.

## 1. Phenotypic Plasticity in Insects

Phenotypic plasticity allows individuals to alter their behaviour, physiology or morphology in response to an environmental cue [4,5,6]. The suggestion that plasticity has a significant role in evolution was first made by West-Eberhard [4,7], and plasticity has since been hailed as a possible rapid-response mechanism allowing animals to adapt to changing or fluctuating environments [4,5,6,7]. Phenotypic plasticity is common among insects, is encoded in the genome, is ecologically relevant and may influence species’ responses to ecological change facilitating adaptation [8].

Phenotypic plasticity is defined as the ability of an individual genotype to produce different phenotypes when exposed to environmental cues and plasticity can affect the behaviour, biochemistry, physiology or development and morphology of an organism [9]. Phenotypic plasticity is widespread amongst both plants and animals, including insects. Well-known examples of phenotypic plasticity in insects encompass morphology (e.g., the seasonal variation in eyespot patterns in the butterfly, *Bicyclus anynana* [10], and the size of horns in the dung beetle, *Onthophagus taurus* [11]); behaviour (e.g., responses to sexual rivals in, *Drosophila melanogaster* [12]) and plasticity in parental behaviour in the burying beetle, *Nicrophorus vespilloides* [13]; learning [14]; and immunity [15].

**Figure 1:**
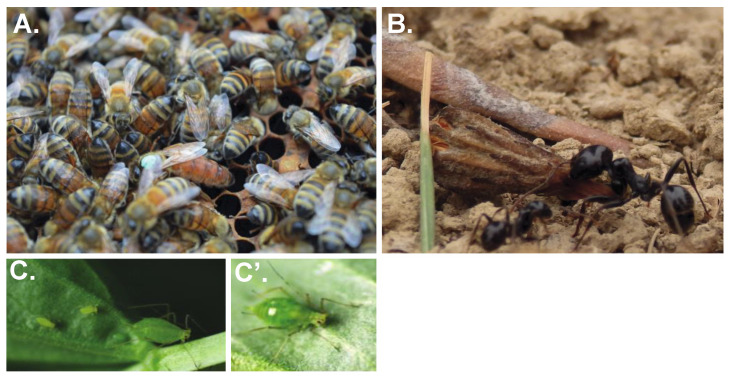
Phenotypic Plasticity in Insects. Insects exhibit remarkable examples of phenotypic plasticity, termed polyphenisms. (**A**) Queen (pictured with a green mark on her thorax) and worker honeybees (*Apis mellifera)* are both female and derived from the same genome. Queens and workers differ in physiology, behaviour and lifespan and these differences are established due to differential nutrition during early development (reviewed in [19]). (**B**) Worker (left) and soldier (right) castes of the European Harvester Ant *(Messor barbarous*). Both workers and soldiers are female but it is not yet known what environmental cue influences the development of the two castes. (**C**) The pea aphid (*Acyrthosiphon pisum*), like most aphids, can reproduce asexually ((**C**), viviparously) or sexually ((**C’**), oviparously) dependent on day-length and temperature (reviewed in [20]). Remarkably, the pea aphid genome not only encodes two different ways of reproducing but also two different ways of undergoing early embryonic development [21].

Perhaps the best-known examples of plasticity come from special cases of plasticity in insects known as polyphenisms, where rather than having a continuous difference in a phenotype (e.g., body size or timing of a behavioural response), individuals have discrete classes of phenotypes. Well-known examples include the queen-worker and worker-worker caste polyphenisms of social insects (Figure 1), gregarious forms of locusts and the wing and reproductive polyphenisms of aphids (Figure 1) [16,17,18].

## 2. Epigenetics and Plasticity

**Figure 2:**
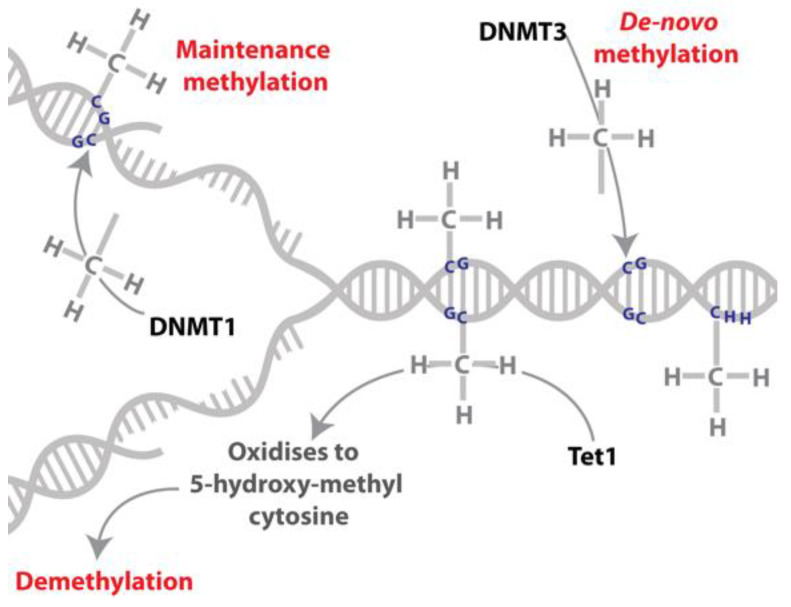
DNA methylation and demethylation. DNA can be reversibly modified by the addition of a methyl group on a cytosine residue. The addition of the methyl group to this residue is catalysed by DNA methyltransferases (DNMT3 catalyses de novo DNA methylation while DNMT1 has been characterised as a maintenance methyltransferase). DNA can be demethylated by the oxidation of the methylated cytosine to 5-hydroxy-methylcytosine, which is catalysed by the TET (ten-eleven-translocase) family of proteins.

Epigenetic mechanisms encapsulate any heritable change of an organism’s phenotype that does not change its DNA sequence. Epigenetic mechanisms include cytosine methylation (commonly referred to as DNA methylation, Figure 2), histone tail post-translational modifications (PTMs), non-coding RNAs and potentially the way that chromatin is organised within the nucleus of the cell [22,23,24,25]. Epigenetic mechanisms regulate gene expression patterns and can define and alter cell and organism phenotypes. The epigenome (all the epigenetic modifications of the genome of an organism) is environmentally responsive in a range of organisms, including insects (reviewed in [26,27]) and has been proposed as a major mechanism mediating genome responses to environmental change directly. The extent to which an organism can respond to current and future climate extremes via epigenetic mechanisms may influence its capacity to cope with global change [28].

The link between epigenetics and phenotypic plasticity in animals was most famously established for the ‘*Agouti viable yellow*’ mouse where genetically identical mice exhibit differences in coat colour ranging from yellow to pseudoagouti. These differences are due to differential DNA methylation of a transposable element in the agouti coat colour gene locus [29]. Importantly, DNA methylation and coat colour in these mice can be affected by maternal diet [30,31,32] stimulating a new research field focussing on epigenetic mechanisms, especially DNA methylation, being a link between phenotypic plasticity and environmental cues (reviewed in [22,33,34]). Here, we review the evidence that DNA methylation is a mechanism that underpins the phenotypic plasticity of insects.

## 3. DNA Methylation

Methylation of cytosine residues (DNA methylation) has received particular attention from the field, in large part because of the well-established techniques used to assess cytosine methylation levels [35].

DNA methylation occurs in plants, animals, fungi and bacteria [36,37], and involves the addition of a methyl group to a cytosine residue (Figure 2). In animals the transfer of this methyl group is catalysed by DNA methyltransferase enzymes; DNA methyltransferase 3 (DNMT3) is responsible for the majority of de novo DNA methylation and DNA methyltransferase 1 (DNMT1) is responsible for maintaining the DNA methylation marks through cell divisions (maintenance methyltransferase; [38,39]). DNA methylation is reversible and the ten-eleven-translocase (TET) family of enzymes have a role in this process by converting 5-methylcytosine to 5-hydroxymethylcytosine [40,41], which can, in mammals, be subsequently oxidised to 5-formyl- and 5-carboxyl-cytosine [42]. 5-hydroxymethylcytosine is present in insect genomes at appreciable levels, e.g., [43,44,45], but the presence of 5-formyl- and 5-carboxyl-cytosine in insects and the function of these modifications remain unknown. In addition, it is proposed that DNA replication in the absence of maintenance of DNA methylation could result in passive demethylation [42]. Replication independent removal of methylated cytosines may also be achieved via multiple rounds of oxidation by TET and DNA Glycosylase (DG) family of enzymes or via deamination by activation induced deaminase (AID) enzymes with repair by base excision repair machinery [42,46]; Figure 2.

Because DNA methylation is linked to phenotypic variation in some mammalian species, DNA methylation has been assumed to be a key pathway for mediating environmental responsiveness across all animals.

## 4. What Are the Functions of DNA Methylation in Insects?

Unlike mammals, where DNA methylation of promoter regions represses gene expression [47], DNA methylation in insects, with the odd exception [48,49], predominantly occurs within genes [36,39,50]. The absolute levels of DNA methylation vary between insect species [49,51] and DNA methylation tends to occur in genes that are highly conserved [49,52] and annotated with “housekeeping” functions [49,52]. DNA methylation is more often found in genes that are present as single copies in the genome, with decreased methylation in duplicated genes [53]. This pattern of methylation implies different functions of methylation in insects compared with mammals.

In mammals, DNA methylation regulates gene expression [47], but does it do so for insects?

In insects, high levels of methylation seem to be associated with genes that are conserved, broadly and highly expressed, rather than genes that are expressed in a tissue or specific manner [49,51,54,55,56,57,58,59,60]. At the level of individual genes, however, this association is weak and highly methylated genes can have low expression [49,58].

The complex relationship between DNA methylation and gene expression is reflected by several studies across a range of insects, including beetles [61], ants [57,59] and bees [62,63,64,65], that show no association between differences in DNA methylation and gene expression. In the silkmoth, *Bombyx mori*, embryonic diapause and development are associated with substantial changes to DNA methylation across the genome, affecting over 4500 genes. However, only a small proportion of those genes were differentially expressed (<10%) and these were not consistently up or down-regulated [66]. Similar conclusions were drawn in a study of metamorphosis in the tobacco hornworm, *Manduca sexta* [67]. Differences in DNA methylation were not associated with differential gene expression in honey bees that become more aggressive after an intruder test [68].

Some studies have, however, hinted at an association between gene expression and differential methylation; for example: in the burying beetle, *Nicrophorus vespilloides*, 51% of genes that were differentially expressed in the brain after one generation of selection for no parental care were also associated with differential methylation. However, this relationship was not present after 30 generations of selection [69]. DNA methylation may also mark a sub-set of genes allowing them to be ‘poised’ for later gene expression, as has been documented for a histone modification in the honeybee [70]. Two recent studies in honeybees have hinted that changes in DNA methylation may precede changes in gene expression [68,71]; although, neither showed a direct or causal association between differences in DNA methylation and differences of gene expression.

Critical to our understanding of the function(s) of DNA methylation in insects is to determine if there is a causative link between DNA methylation and gene expression. Despite this, few studies have used functional genetics to assess the role of DNA methylation in insects. Knock-down of *Dnmt1a* in the jewel wasp, *Nasonia vitripennis*, demonstrated that a reduction in DNA methylation was generally associated with decreased gene expression [72]. A similar approach in the migratory locust *Locusta migratoria* showed that reducing *Dnmt3* levels resulted in the differential expression of 346 genes in the brain, most down-regulated [73], but without measuring methylation directly. Reduction in the expression of *Dnmt3* in the abdominal fat body of honey bees (*Apis mellifera)* resulted in differential expression of over 2500 genes [74]; however, it is not clear if these genes followed a general pattern of either up-or down-regulation. Experimental knockdown of *Dnmt1* in the milkweed bug, *Oncopeltus fasciatus*, showed significant alterations to the patterns of DNA methylation but few genes were also differentially expressed [75].

It has been proposed that DNA methylation within introns may regulate alternative splicing of mRNA transcripts [54,58,64,74,76,77], possibly by mediating access to DNA binding factors or by slowing RNA polymerase [78,79]. However, recent analyses of whole-genome data sets have shown that there is no consistent relationship between DNA methylation and alternative splicing in a range of insects [49,62,80,81].

DNA methylation may also influence gene expression by modifying transcription factors’ affinity for *cis*-regulatory elements [82] and may stabilise transcription, enhance the fidelity of transcription by reducing transcriptional variability of gene expression [83,84] or prevent initiation of transcription due to cryptic sites within genes [85].

However, recent functional analysis in the silkmoth, *Bombyx mori*, indicates that DNA methylation near the transcriptional start site regulates gene expression by modulating histone modifications (specifically H3K27 acetylation) and requires the highly-conserved methyl-binding protein, MBD2/3, and the Tip60 complex [86]). This study, therefore, provides evidence for a direct mechanistic link between gene expression and DNA methylation in *B. mori*. However, given the complex and combinatorial nature of histone modifications, there may be unknown subtleties that explain the variable effect of DNA methylation on the expression of individual genes. Further, it will be essential to determine whether the link between DNA methylations, histone modifications and gene expression are conserved amongst insects.

Although there are a few exceptions, our review of the available studies using insect species showed no evidence of a consistent association between differential DNA methylation and differential gene expression. Further, whether a direct mechanistic link between DNA methylation and gene expression across insects still needs to be established.

## 5. How Does DNA Methylation Respond to Environmental Change and Phenotypic Plasticity?

That we are unable to draw broad or universal conclusions about the relationship between DNA methylation and gene expression is surprising and implies that we either poorly understand the function of DNA methylation, or that it carries out a range of activities, some of which may be taxon-specific. But can we approach the question another way? When significant gene expression changes occur in the genome, such as plastic responses to the environment or events, such as caste development, what happens to DNA methylation?

While studies of genome-wide DNA methylation patterns have not indicated a key role in regulating developmental plasticity, many studies have shown that DNA methylation in insects changes under a variety of environmental conditions, such as parasitism [87], photoperiod [88] and immune challenges, e.g., [71].

The role of DNA methylation in phenotypic plasticity was first experimentally tested in the honey bee (*Apis mellifera).* Knockdown of the de novo methyltransferase *Dnmt3* mRNA by RNAi reduced the ability of larval honey bees to develop into workers [89]. DNA methylation was interpreted as having a key and causative role in caste specification in the honey bee. The authors’ demonstrated that knockdown of *Dnmt3* was associated with some changes in gene expression but only examined differences of DNA methylation at a single locus, *Dynactin p62*. Subsequent studies have demonstrated differences in DNA methylation and DNA methylation dynamics in the larvae of queen and worker castes in the honeybee [90,91,92]. However, for these experiments, the earliest sampling period was three days after the larvae hatch. At this time juvenile hormone levels are already significantly different between queen and worker larvae, and indeed this sampling period corresponds with the peak of juvenile hormone levels in queen larvae (reviewed in [93]). Juvenile hormone is a global regulator of gene expression, and at this sampling period of larval development there are more than 2500 genes differentially expressed [94]. This means that differences in DNA methylation observed around this sampling period could be a consequence of whole-scale changes in gene expression and not causative of them. Importantly, this sampling period also occurs after larvae are already committed to a queen or worker fate [95], again suggesting a correlative rather than a causative association between DNA methylation and caste. The role of DNA methylation in caste specification could be resolved further by sampling at earlier time points, e.g., between 6 h of larval development where differential gene expression between queen and worker destined larvae is first detected [94] and also at 60 h of larval development when larvae are committed to queen or worker fate [95]. In light of this, and the discovery of non-methylation functions of the DNMT genes [75,96,97], the role of DNA methylation in caste specification in the honeybee warrants re-investigation.

As a result of the initial work by Kucharski and colleagues [89], further research into the role of DNA methylation in phenotypic plasticity has focused on caste polyphenisms in the Hymenoptera (reviewed in [34]). Differences between castes are usually established early in insect life (during larval or nymphal development). Unfortunately for many species, we do not know the critical times in which caste is induced or the environmental cues that trigger caste specification. Many studies of DNA methylation in these species have focussed on adults, long after the events of caste specification have occurred. Drawing general conclusions from these studies is difficult, as they focus on species that differ in the morphological differentiation between queens and workers, use a range of techniques to assess DNA methylation (reviewed in [34]) and focus on adults. Some studies in ants, wasps and termites report systematic differences in DNA methylation between queen and worker castes [58,59,77]. In comparison, no differences in DNA methylation between castes of other species, such as the paper wasp, *Polistes canadensis*, and the dinosaur ant, *Dinoponera*, were detected [81]. Further, Libbrecht and colleagues [57] demonstrated that earlier studies’ results reported that differences reported between castes were likely driven by very low sample sizes [57] or pooling of samples [34].

These studies may also be confounded by recent reports in ants [57], bumblebees [62] and honey bees [65] (Duncan and Dearden, unpublished data) in which DNA methylation varies significantly between colonies. This indicates that there is a genetic effect on DNA methylation, that colony environment influences DNA methylation, or that DNA methylation is not environmentally responsive in adults (it may be set when individuals are developing). If this ‘colony-level’ effect is a common phenomenon amongst insects, this would be a major confounding factor in many DNA methylation studies.

Some studies in eusocial insects may also be confounded by differences in the rates of development and ageing between eusocial queens and workers; development time often differs between queens and workers, and eusocial queens live longer than eusocial workers [98]. It is thus difficult to know if differences in methylation are due to caste or to age.

Differences in DNA methylation between castes could be a consequence of different caste biology rather than a cause of caste specification. As a result of these seemingly conflicting results and the limitations in the experiments, the role of DNA methylation in caste specification is still somewhat controversial.

In eusocial species one female, the queen is responsible for most reproduction. In some species, such as the bumblebee and honeybee, workers still retain some capacity to reproduce. In honey bees, worker reproduction is triggered by the loss of the queen and her pheromone (queen mandibular pheromone, QMP). Reproductive plasticity in honeybees has been associated with epigenetic changes, including chromatin modifications and widespread changes in gene expression [70,99], but a recent study showed that there were no significant differences in DNA methylation in the ovaries or brains of worker bees as a result of reproductive plasticity [65]. Bumblebees showed some caste-specific differences in brain DNA methylation [62], consistent with previous experimental manipulations using a chemical inhibitor of DNA methylation [100], but this was not linked with gene-expression changes in the brain.

There is a relative paucity of studies examining the role of DNA methylation in other examples of insect phenotypic plasticity (reviewed in [101]). The studies that have been carried out do not indicate a clear role for DNA methylation in phenotypic plasticity. Dispersal plasticity occurs in locusts, where individuals shift between a solitary to a gregarious phenotype in response to crowding. This has been associated with differences in DNA methylation in the central nervous system of the desert locust (*Schistocerca gregaria*). This polyphenism is also associated with differential expression of genes involved in DNA methylation [102] and functional studies in the migratory locust *Locusta migratoria* have indicated that *Dnmt3* might be required for some aspects of dispersal plasticity in this species [73], but this has not been linked to specific changes to the methylome. Plasticity of horn size in the dung beetle *Onthophagus gazella* has also been examined*,* with little evidence for DNA methylation changing with nutritional plasticity [103]. There are substantial differences in DNA methylation associated with photoperiod and embryonic diapause in *N. vitripennis*. Reduction in *Dnmt* gene expression by RNAi or blocking DNA methylation with a chemical inhibitor does partially block this response [88]. However, further work is required to determine which tissues and regions of the genome are affected and to rule out any non-DNA methylation functions associated with the DNA methyltransferase enzymes.

Behaviour is thought to be intrinsically more plastic than morphology and there are many examples of plastic behaviour of insects. The role of DNA methylation during behavioural plasticity has been assessed broadly among insect taxa stemming from early reports of DNA methylation differences among castes of eusocial insects [54,58,89,92] and the desert locust [104]. During temporary transitions into parental care, differences in DNA methylation were not detected among differentially expressed genes in the burying beetle *Nicrophorus vespilloides* [61] or the clonal raider ant [57]. This pattern also holds for more permanent changes among castes that are accompanied by a suite of behavioural differences of eusocial insects: honey bees [65]; carpenter bee, *C. calcarata* [64]; the paper wasp, *Polistes canadensis*; or the dinosaur ant, *Dinoponera* [81]; and the ant, *Formica exsecta* [59]. Some eusocial species maintain castes without DNA methylation [105]. Aggression differences of honeybees after an intruder test are accompanied by a few methylation changes, but those are not associated with gene expression differences [68]. However, some evidence does exist to link methylation to gene expression differences associated with different behaviours. Mashoodh and colleagues [69] showed differentially methylated genes were overrepresented among differentially expressed genes for the burying beetle, *Nicrophorus vespilloides*, with some different parental care regimes, but this was not true of all of their treatments.

**Figure 3:**
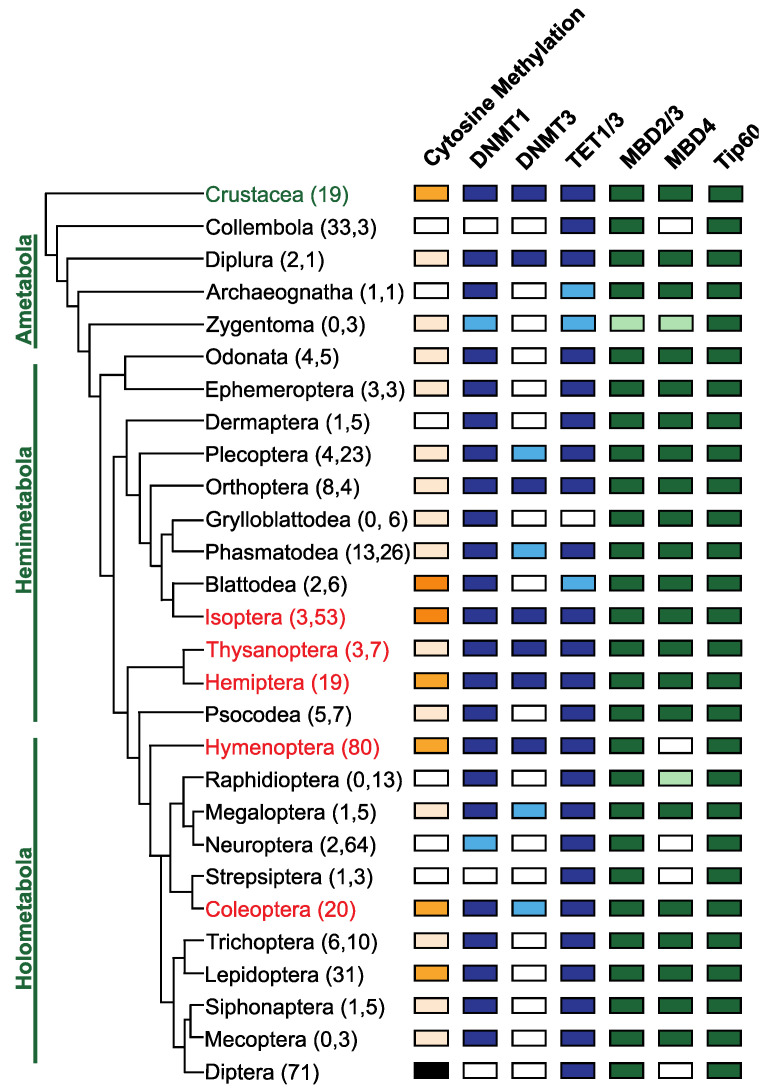
Conservation of DNA methylation and genes involved in DNA methylation in Insects. The phylogenetic relationships between insect orders are based on Misof et al. [106], and orders where eusociality is present are indicated in red. The number of genomes and transcriptomes interrogated to determine the presence or absence of genes involved in DNA methylation are indicated in parentheses following the name of the insect order (data supplied as Supplementary Table S1). The presence of DNA methylation in insect orders was obtained from published data [49,51,52]. Orders where the presence of DNA methylation is confirmed by experimental evidence (whole genome bisulfite sequencing) are indicated in dark orange, orders where DNA methylation has been inferred based on analysis of the CpG[o/e] are indicated in apricot. Orders where DNA methylation is absent, and this has been demonstrated experimentally, are coloured black and those where the absence is inferred based on analysis of CpG[o/e] are coloured white. The presence or absence of genes involved in DNA methylation was determined via BLAST [107] analysis of publicly available reference genomes assemblies and transcriptome shotgun assemblies (TSA). Genes directly implicated in DNA methylation are coloured in blue, while genes involved in interpreting DNA methylation [86] are indicated in green. Orders where less than 50% of the sampled species have an identifiable homolog are indicated by a lighter colour, indicating some variation in gene conservation within orders. DNMT1 is found in species belonging to all insect orders except in Collembola, Strepsiptera and Diptera. In contrast, DNMT3 was only identified in a subset of insect orders. TET1, putatively involved in DNA demethylation, is conserved in all insect orders except the Grylloblattodea. TET1 is present in insect orders that are missing DNMT1/DNMT3 and may be evidence for a DNA methylation independent function for this gene. MBD2/3 and Tip60 are highly conserved, but MBD4 has been lost at least five independent times in the evolution of the insects in the lineages giving rise to the Collembola, Hymenoptera, Neuroptera, Strepsiptera and Diptera.

Perhaps the key to understanding DNA methylation in insects is that it is not ubiquitous. Several insect lineages, including Diptera, some Lepidoptera, Coleoptera and Hymenoptera, have lost one or both of their DNA methyltransferase genes and have low or no detectable DNA methylation in their genomes (Figure 3) [51]. Yet some species, such as *Drosophila melanogaster*, lack substantial DNA methylation and show marked examples of plasticity [12]. This implies that if there is a link between plasticity and DNA methylation, it is not universal.

## 6. Non-Methylation Functions of the DNMT Genes

Some insects do not have detectable DNA methylation but do retain a copy of the DNMT1 maintenance methyltransferase gene (Figure 3) [51], raising the question of what these enzymes are doing in these species. In the red flour beetle, *Tribolium casteneum*, RNAi demonstrates a critical role for *Dnmt1* in oogenesis and embryogenesis [96]. This phenotype is also seen in the Hemipteran milkweed bug, *Oncopeltus faciatus* [75,97]. This suggests that the DNMT enzymes may have ancestral or conserved molecular functions independent of DNA methylation. It also raises the possibility that the role of DNMT3 in honeybee caste specification may relate to a non-DNA methylation activity of this gene [89].

## 7. How Might We Demonstrate a Role of DNA Methylation as a Causative Factor in Plasticity?

In insects, the role(s) of DNA methylation remains unclear. While DNA methylation has been associated with the outcomes of plasticity, there is no unequivocal evidence for a causal role for this epigenetic mark in phenotypic plasticity. With evidence for roles of methylation enzymes outside DNA methylation, we now have a whole raft of possible functions and activities for DNA methylation systems in insects that have not been well studied. If we are to be able to understand if DNA methylation is a key mediator of plasticity in insects, we need to fill crucial gaps in our knowledge.

**Where?**

DNA methylation patterns across the genome are often assessed in bulk tissues, such as the whole brain, whole ovary, or even whole body. These kinds of analyses are problematic because each cell has its own developmental and differentiation history, which leads to changes in gene expression, and presumably, changes in DNA methylation. By using whole tissues containing different cell types, we knowingly homogenise those DNA methylation signals, blunting our analysis and masking differences in DNA methylation (as each cell in the sample contributes an equal amount of DNA to a sample). This is particularly problematic if, as seems very likely, plasticity leads to different ratios of cell types in a tissue. If methylation is fixed within cell types, then bulk analysis will point to differential methylation and imply a regulatory role for DNA methylation, when in fact the differences in DNA methylation merely reflect different numbers of cells of particular types, e.g., [108].

The response to an environmental cue could feasibly come from a small number of signalling cells (for example the prothoracic gland or corpora allata) expressing signalling molecules or circulating hormones. Unless we examine DNA methylation patterns cell by cell, we may miss the crucial cell type, and therefore miss crucial DNA methylation changes.

We advocate for more in-depth cell biology to understand which cells in insects change their methylation patterns in response to environmental events and which cell populations drive, or are affected by, plasticity (refer to question 2 below).

**When?**

Many studies target tissues in adults for studies of DNA methylation and plasticity. However, as discussed for caste specification, if DNA methylation is underpinning plasticity, we would expect it to act at a specific stage of development or following a specific environmental cue. It is possible that DNA methylation changes in response to environmental cues may be transient and tissue-specific. Even in many of the ‘textbook’ examples of phenotypic plasticity, we are only beginning to understand how the environmental signal is received, how this signal is sent to the affected tissue and how the change in phenotype is implemented. To interrogate the role of DNA methylation in phenotypic plasticity, we need to have a much better understanding of the fundamental processes that underpin phenotypic plasticity. For example, reproduction in the honeybee worker is repressed by a queen and her pheromone, but when the pheromone is removed, what tissue (brain, antennae, ovary or fat body) is it appropriate to examine changes in DNA methylation? At what point after queen mandibular pheromone is removed is the critical period to assess DNA methylation? We currently lack an in-depth understanding of the physiology, neurobiology and developmental aspects of plasticity in many species, and this knowledge is crucial to identify critical sampling periods and tissues of interest for epigenetic and other mechanistic analyses.

**How?**

To demonstrate causative roles of DNA methylation for phenotypic plasticity, we need to understand how they might be linked. Noting changes in DNA methylation associated with alternative phenotypes (summarised in Section 5) is not enough. We can only directly demonstrate the role of a specific change in DNA methylation through highly sophisticated experimental approaches that manipulate levels of DNA methylation and that have predictable consequences to phenotypes.

Few studies have addressed the function of DNA methylation in insects (summarised in Section 5). Those studies that assess the function of DNA methylation rely on RNA interference (RNAi) or pharmacological inhibition of DNA methylation. RNAi reduces but does not fully eliminate the expression of the gene of interest, and the level of reduction can be variable. This can result in a range of phenotypes that may be difficult to interpret and may be confounded by functional redundancy of the targeted gene. In *D. melanogaster*, the UAS-GAL4 system allows RNAi to be targeted to specific tissues [109] and also allows fine temporal control of the gene knockdown [110]. However, the UAS-GAL4 system has not been widely established in species beyond *D. melanogaster.* Moreover, RNAi has variable efficacy in different species as well as within species at different life stages and in different tissues [111,112].

The other commonly used approach is to use pharmacological inhibitors of DNA methylation (e.g., 5-Azacytidine, Zebularine). These compounds inhibit DNA methylation by binding irreversibly to DNMT1. They all need to be present in cells at the S phase of the cell cycle to have an effect, which means that tissues undergoing rapid cell division may show preferential effects of these inhibitors. Treatment with these inhibitors has also been reported to cause hypermethylation of some loci [113], making interpretation of phenotypes and effects on gene expression difficult.

These approaches do not, however, allow the fine-scale analysis the field needs. Rather than affecting DNA methylation in general, the ability to reversibly methylate, or demethylate specific sites in the genome, ideally in specific cells, is needed to directly test the role of the DNA methylation itself on phenotypic plasticity. This kind of manipulation sounds fantastical; however, this is now possible, at least in human cell-culture using the CRISPRoff and CRISPRon system [114]. This system uses a guide RNA to target a modified Cas9 enzyme to a specific region of the genome and induce methylation which can be reversed. This kind of tool, once linked to cell-type specific analysis, such as the UAS-GAL4 system used extensively in *Drosophila* [109], provides the potential to categorically demonstrate the link between DNA methylation of specific loci in specific cell-types, and phenotypic plasticity.

## 8. Conclusions

Studies to date have not demonstrated an unequivocal role for DNA methylation in phenotypic plasticity. However, there are hints that DNA methylation may function in some examples of phenotypic plasticity. Studies to date have been hampered by technological and analytical limitations that have made it difficult to mechanistically link an environmental cue to changes in DNA methylation, gene expression and ultimately phenotype. As a field, we need to ensure that our experimental approach is fit for purpose and will provide unequivocal evidence either for or against the role of DNA methylation in insects (Box 1). New approaches, which facilitate higher sensitivity allowing cell-type specific analysis as well as tools that facilitate the manipulation of DNA methylation, are set to give us unprecedented insight into the cellular role of DNA methylation in insects and the role, if any, of DNA methylation in mediating phenotypic plasticity.

Box 1 **Experimental standards in methylation experiments.**

Our understanding of methylation in insects is hampered by our differing approaches, experimental standards, and lack of tools. We propose that convincing evidence for the function of DNA methylation in insects and, more specifically in plasticity, requires specific predictions, planning and experimentation. We provide this box to help researchers plan such experiments.

A Measurement of DNA methylation:
The gold standard for measuring DNA methylation is whole genome bisulfite sequencing (WGBS) [115]. This technique is well established and involves chemical treatment of the DNA, converting unmethylated cytosines to uracils that are then detected after sequencing and comparison to the reference genome. Alternative sequencing technologies, such as the Oxford Nanopore, that do not rely on chemical modification of the DNA, are becoming available and are rapidly expanding this field [116] and may allow for the simultaneous detection of 5-methylcytosine and its oxidised derivatives, such as 5-hydroxymethylcytosine.
B Experimental design:
Studies need adequate statistical power as a lack of replication has been associated with spurious associations between DNA methylation and differential expression [57]. Biological replicates should consist of as few individuals as possible. If strong genetic differentiation is suspected among samples, this will increase the noise in the experiment, making it more difficult to detect biological signals, and if samples are genetically similar, this may predispose for false positives. Genetically related individuals should be considered as ‘technical’ rather than ‘biological replicates’ to be assured that the differences identified are biological and not simply genotype-specific (e.g., among different colonies of honey bees [65]). In practice, for social insects, this means that one hive/colony is a biological replicate.
C  Analysis of DNA methylation and comparisons with gene expression:
Careful consideration needs to be given to analysis to ensure their appropriateness for the question asked. Pipelines often use models made for methylation analysis of mammals rather than insects. In mammals DNA methylation is dense enough to approximate a continuous trait (e.g., 76% of cytosines are methylated for house mice, while only 0.4–1.5% are methylated for honey bees). A recent pipeline (BWASP/R) has been developed that is tailored to species with low-levels of DNA methylation [117] and use of a standardised approach specific to insects will certainly facilitate our understanding of the role of DNA methylation in phenotypic plasticity. In species with low levels of methylation, the methylation status of cytosines should be determined on a case-by-case basis using a statistical test against the genomic background. % difference of methylation status has no biological interpretation as individual cytosines are either methylated or not. Gene-level methylation should then be assessed by a summary metric of % cytosines methylated contained within the gene body or within biologically relevant and consistent gene regions. Comparisons between DNA methylation and gene expression need to be modified to account for the fact that differences in DNA methylation may have different functions depending on where in the gene the methylation mark is located and these approaches should also be standardised to facilitate comparison between species and studies. Network based approaches can also be employed as DNA methylation can exhibit network structures just as gene expression can, and these can be preserved in gene co-expression networks [59].
D  Cell-type/Tissue-specific analysis:
Phenotypic plasticity experiments have often been carried out on whole tissues or bodies that contain multiple cell types. This masks differences in DNA methylation that occurs only in a subset of cells. There have been some approaches to examine ‘bulk WGBS’ data from mammals to identify underlying cell-type-specific patterns [118]. However, such approaches are unlikely to be useful at present in insects as the patterns of DNA methylation differ substantially from mammals. Cell-type-specific assessment of DNA methylation is possible in other animals, using either cell-sorting or single-cell analysis, and could be adapted for use in insects. In the short-term, identification of the smallest amount of and most specific tissues that are affected by plasticity and possibly even regions of those tissues (e.g., mushroom bodies for behavioural plasticity) should be targeted for studies of DNA methylation.
E  Functional analysis:
Many studies that report differences in DNA methylation associated with plasticity lack a definitive functional experiment. In non-model systems, the tools available to do this are limited, and RNA interference or treatment with pharmacological inhibitors is necessary. Although there are limitations to these approaches (as discussed in Section 7), these are still powerful approaches to examine gene function, but this needs to be linked *a priori* with discrete phenotypes of interest. Of technical importance is to ensure a meaningful level of reduction of the *Dnmt *mRNA in the tissue of interest at the appropriate timing and combine this with an assessment of treated individuals to respond plastically. Further, this needs to be combined with techniques (such as WGBS) to demonstrate the reduction in DNA methylation and to locate specific regions of the genome that have altered methylation in response to the treatment and RNA-seq to be able to test the link with changes in gene expression. Although the sequencing we suggest does increase the cost and complexity of any experiments, we believe that these links are necessary to fully interpret and link DNA methylation with gene expression and plasticity. Any experiment that does not demonstrate core mechanistic links (*Dmnt* gene expression reduction leading to reduced DNA methylation leading to altered gene expression in the appropriate tissue/cell type at the appropriate time leading to predicted changes of phenotypic plasticity) has its ability to speak about causality reduced. Other techniques to manipulate gene expression or DNA methylation are becoming available and should be considered.
F  Establishing the consequence of specific changes to the methylome:
The ultimate test of the effect of DNA methylation is to be able to experimentally manipulate DNA methylation at a particular locus and show that there are predictable downstream consequences for phenotypic plasticity. Tools, such as the CRISPRoff and CRISPRon system [114], can be investigated for this purpose for insects.

## Data Availability

All data is presented in this manuscript.

## Supplementary Materials

The following supporting information can be downloaded at: https://www.mdpi.com/article/10.3390/insects13020110/s1, Table S1. The number of genomes and transcriptomes in insects to determine the presence or absence of genes involved in DNA methylation.
